# Intraoperative Ultrasound in Hepatic Oncology Surgery: A Narrative Review of Its Impact on Surgical Strategy and Oncologic Outcomes

**DOI:** 10.3390/cancers18142309

**Published:** 2026-07-17

**Authors:** Cosmin Nicolescu, Catalin Dumitru Cosma, Marian Botoncea, Adrian Bartoș, Călin Molnar

**Affiliations:** 1 Faculty of Medicine, George Emil Palade University of Medicine, Pharmacy, Sciences and Technology of Târgu Mureș, 540139 Targu Mures, Romania; cosmin.nicolescu@umfst.ro (C.N.); marian.botoncea@umfst.ro (M.B.); calin.molnar@umfst.ro (C.M.); 2 General Surgery Clinic No.1, County Emergency Clinical Hospital of Târgu-Mureș, 540136 Targu Mures, Romania; 3 Institute of Advanced Studies in Science and Technology, Babeș-Bolyai University, 400347 Cluj-Napoca, Romania; bartos.adi@gmail.com; 4 Faculty of Medical and Health Sciences, Babeș-Bolyai University, 400347 Cluj-Napoca, Romania; 5 Regina Maria Hospital, 400117 Cluj-Napoca, Romania

**Keywords:** intraoperative ultrasound, contrast-enhanced intraoperative ultrasound, hepatic oncology surgery, hepatocellular carcinoma, colorectal liver metastases, precision liver surgery, artificial intelligence, image-guided surgery

## Abstract

**Background/Objectives:** Intraoperative ultrasound (IOUS) has become an integral component of modern hepatic oncology surgery, providing real-time imaging guidance during liver resections for hepatocellular carcinoma, colorectal liver metastases, and other primary or secondary hepatic malignancies. Despite substantial improvements in preoperative imaging modalities, occult lesions, disappearing metastases after chemotherapy, and complex vascular relationships continue to represent major intraoperative challenges. This structured narrative review aimed to evaluate the contemporary role of IOUS in hepatic oncology surgery, with particular emphasis on contrast-enhanced intraoperative ultrasound (CE-IOUS), minimally invasive liver surgery, navigation-assisted hepatectomy, and emerging artificial intelligence-based technologies. **Methods:** A structured literature review was conducted using PubMed/MEDLINE, Scopus, Web of Science, and Google Scholar databases. Peer-reviewed studies, international guidelines, consensus statements, systematic reviews, and technological reports addressing IOUS applications in liver surgery were analyzed. Particular focus was placed on studies evaluating lesion detection, intraoperative strategy modification, disappearing colorectal liver metastases, parenchymal-sparing hepatectomy, laparoscopic and robotic liver surgery, navigation systems, augmented reality integration, and AI-assisted imaging technologies. **Results:** Contemporary evidence demonstrates that IOUS continues to significantly influence intraoperative decision-making despite advances in magnetic resonance imaging and multidetector computed tomography. CE-IOUS improves the detection of occult hepatic lesions and residual disease after systemic chemotherapy, particularly in disappearing colorectal liver metastases. IOUS-guided anatomical and parenchymal-sparing resections contribute to the preservation of functional liver parenchyma while maintaining oncologic radicality. In minimally invasive liver surgery, laparoscopic ultrasound remains essential for lesion localization and vascular mapping. Recent developments integrating navigation systems, augmented reality platforms, and AI-assisted image recognition suggest a progressive transition toward digitally integrated precision liver surgery. **Conclusions:** IOUS remains a cornerstone of modern hepatic oncology surgery and continues to evolve from a localization tool into a comprehensive platform for precision-guided liver resection. The integration of CE-IOUS, navigation technologies, and artificial intelligence may further enhance intraoperative accuracy, oncologic safety, and individualized surgical planning in the future.

## Simple Summary

Liver malignancies, particularly hepatocellular carcinoma and colorectal liver metastases, remain major challenges in surgical oncology. Despite substantial advances in preoperative imaging, occult lesions, vascular invasion, and chemotherapy-altered metastases may still remain undetected before surgery. Intraoperative ultrasound (IOUS) has therefore become an essential real-time imaging modality in hepatic surgery, enabling accurate lesion localization, vascular mapping, anatomical guidance, and parenchymal-sparing resections. The development of contrast-enhanced intraoperative ultrasound (CE-IOUS), laparoscopic ultrasound, navigation systems, fluorescence-guided surgery, augmented reality, and artificial intelligence-assisted technologies has further expanded its role in precision liver surgery. Contemporary evidence demonstrates that IOUS continues to influence intraoperative decision-making even in the era of high-resolution magnetic resonance imaging and computed tomography. This structured narrative review summarizes the current applications, oncologic impact, and future technological perspectives of intraoperative ultrasound in hepatic oncology surgery.

## 1. Introduction

Both primary and secondary hepatic malignancies continue to exert a heavy oncological burden worldwide and are among the most difficult contemporary surgical oncology challenges [1,2]. Among these, hepatocellular carcinoma (HCC), which causes the majority of cases of cancer death worldwide at present, is hardly surprising. Similarly, colorectal liver metastases (CRLM) represent one of the most common manifestations of advanced colorectal cancer [1,2]. Surgical resection remains the mainstay of treatment with curative intent for selected patients with HCC and CRLM, providing the greatest chance of long-term survival and disease control if complete removal of the tumor is possible [3,4]. Oncological liver surgery, at the opposite end of the spectrum, is technically demanding due to the complicated intrahepatic vascular anatomy, the need to preserve functioning liver parenchyma, and frequent multifocal or occult lesions which preoperative imaging evaluation often incompletely assesses [5,6,7,8]. Over the years, hepatic surgeries have transitioned from major hepatectomies to individualized parenchymal-sparing strategies [9,10]. The shift is aimed at achieving oncological clearance maximally while preserving future remnant liver function [11,12]. Systemic oncological treatments and perioperative care have improved significantly over time, having once been solely limited to palliative care.

In contemporary hepatobiliary surgery, CE-IOUS continues to influence operative strategy by improving lesion characterization, identifying occult disease, and supporting parenchymal-sparing resections [13,14,15,16,17,18,19,20]. Over the past two decades, several studies have reported that CE-IOUS continues to have a major impact on operative strategy and is now considered an essential component for modern hepatic surgery. CE-IOUS has demonstrated substantial value in detecting occult residual disease and guiding targeted resection strategies, and maximizing parenchymal preservation without compromising oncologic radicality [19,20,21,22,23,24]. Parenchymal-sparing hepatectomy is gaining traction increasingly currently. Consequently, IOUS-guided surgery has taken on even greater importance. In real time, it can map intraoperative tumor and portal pedicles, hepatic veins, segmental delineation, and resection margins. The information can facilitate an exact shaping of anatomical resections to the vascular and oncologic anatomy of the patient very often [24,25,26,27,28,29,30,31,32,33,34]. In this way, it is useful to conserve the functional hepatic reserve and avoid unnecessarily major resections. This is particularly true for patients who have bilobar disease and chronic liver disease, or in whom future repeat hepatectomy may be needed.

Over the past decade, hepatic surgery has undergone substantial expansion in both technical complexity and oncologic indications. As a result, there is increased dependence on ultrasound guidance during surgery. Surgeons lack direct tactile feedback with laparoscopic and robotic liver resections. Patients requiring laparoscopic ultrasound include those with smaller lesions, vascular involvement, and lesions located in close proximity to important structures. Gaining more experience with ultrasound-guided parenchymal-sparing surgery, and disappearing metastases in parallel, has strengthened the idea that intraoperative imaging should not be seen as a diagnostic add-on but rather as a driver of surgical strategy [34,35,36,37]. In the same way, international consensus conferences of laparoscopic liver surgery stressed intraoperative ultrasonography’s major role for safe, minimally invasive hepatic resections and complex anatomic operations [38,39,40,41,42,43,44]. Although there has been a significant technological advance in preoperative imaging, there are still significant controversies regarding the current exact role of IOUS, the management of disappearing metastases, the reproducibility of CE-IOUS findings, and the incorporation of emerging navigation and digital technologies into everyday surgical practice. Moreover, the evidence that currently exists is heterogeneous, including retrospective cohorts, prospective observational studies, consensus statements, systematic reviews, and emerging technology reports.

The goal of this narrative review article is to critically assess the current application of intraoperative ultrasound in liver oncologic surgery. It will focus on a number of aspects, including its role in tumor detection, intraoperative decision-making, parenchymal-sparing hepatectomy, minimally invasive liver surgery, and modern precision-guided oncologic strategies. Special focus is given to CE-IOUS, the vanishing of colorectal liver metastases, and technologically assisted intraoperative imaging, which will sensibly influence the framework of precision-guided hepatic surgery in the future.

## 2. Materials and Methods

This manuscript was designed as a structured narrative review intended to provide a comprehensive qualitative synthesis of the contemporary evidence regarding the role of intraoperative ultrasound in hepatic oncology surgery. The review methodology was developed to provide a comprehensive and clinically oriented synthesis of the available evidence while maintaining methodological transparency consistent with contemporary recommendations for narrative evidence synthesis.

A structured literature search was performed using the PubMed/MEDLINE, Scopus, Web of Science, and Google Scholar databases. The literature search included studies published between January 1990 and April 2026 in order to capture both the historical evolution and modern technological developments of IOUS in hepatic surgery. Search strategies combined Medical Subject Headings (MeSH) terms and free-text keywords, including “intraoperative ultrasound”, “contrast-enhanced intraoperative ultrasound”, “CE-IOUS”, “hepatic surgery”, “liver resection”, “hepatocellular carcinoma”, “colorectal liver metastases”, “disappearing liver metastases”, “laparoscopic liver surgery”, “robotic liver surgery”, “navigation surgery”, “augmented reality”, “artificial intelligence”, and “precision liver surgery”. Additional relevant studies were identified through manual screening of reference lists from selected articles, consensus statements, and systematic reviews.

The review primarily included peer-reviewed original articles, prospective observational studies, retrospective surgical cohorts, systematic reviews, meta-analyses, international consensus statements, and clinical practice guidelines addressing the role of IOUS in hepatic oncology surgery. Particular emphasis was placed on studies evaluating intraoperative lesion detection, modification of surgical strategy, vascular mapping, anatomical liver resection, management of disappearing CRLM after chemotherapy, CE-IOUS applications, laparoscopic and robotic liver surgery, navigation-assisted hepatectomy, and digital or artificial intelligence-assisted surgical technologies. Priority was given to studies published in high-impact hepatobiliary, surgical oncology, radiology, and gastrointestinal surgery journals.

Articles not directly related to hepatic oncology surgery, non-English publications without accessible scientific translation, conference abstracts lacking sufficient methodological detail, and purely experimental studies without clinical applicability were excluded from the qualitative synthesis. Given the substantial heterogeneity of the available literature regarding patient populations, imaging protocols, operative techniques, technological platforms, and outcome measures, a quantitative meta-analysis was not performed.

**Figure 1:**
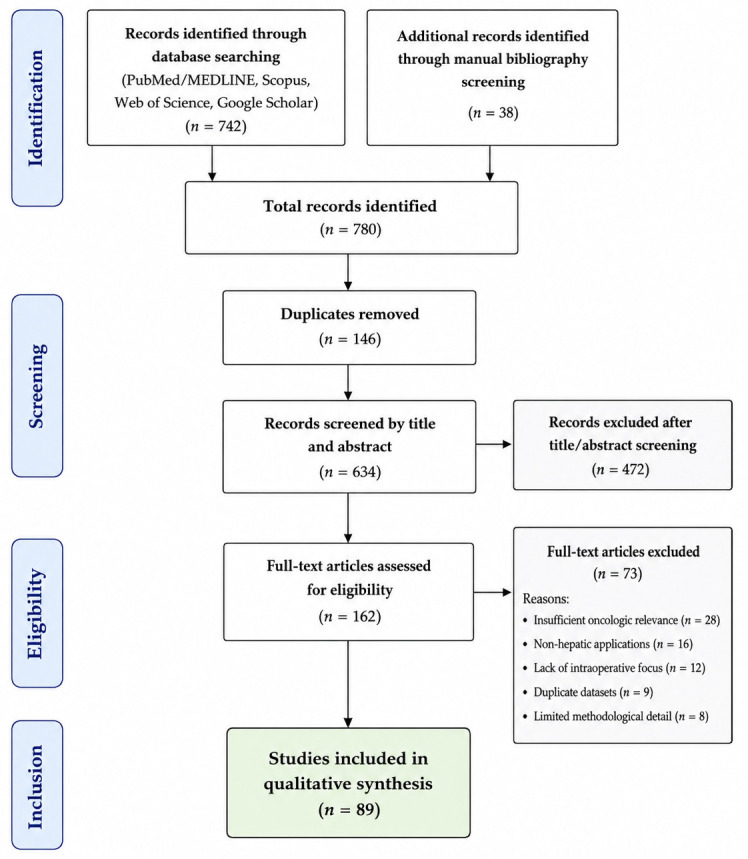
PRISMA-inspired flowchart illustrating the literature identification, screening, eligibility assessment, and qualitative inclusion process used for the structured narrative review of intraoperative ultrasound in hepatic oncology surgery.

Although this review was not conducted as a formal systematic review or meta-analysis, selected methodological principles from the PRISMA 2020 Statement were incorporated to improve transparency and reproducibility of the literature identification and study selection process. Therefore, the PRISMA flowchart should be interpreted as a transparent description of the literature selection process rather than evidence of a formal systematic review methodology (Figure 1).

However, formal risk-of-bias assessment tools, certainty-of-evidence grading systems, and protocol registration were not applied because of the narrative design and the broad technological scope of the review. The final narrative synthesis was structured according to major thematic domains, including the historical evolution of IOUS, lesion detection and intraoperative staging, CE-IOUS applications, disappearing colorectal liver metastases, parenchymal-sparing hepatectomy, minimally invasive liver surgery, navigation systems, augmented reality technologies, and emerging artificial intelligence-based applications in hepatic oncology surgery. Emphasis was placed on integrating classical surgical evidence with contemporary technological innovations in order to critically evaluate the current and future role of IOUS in precision liver surgery.

**Figure 2:**
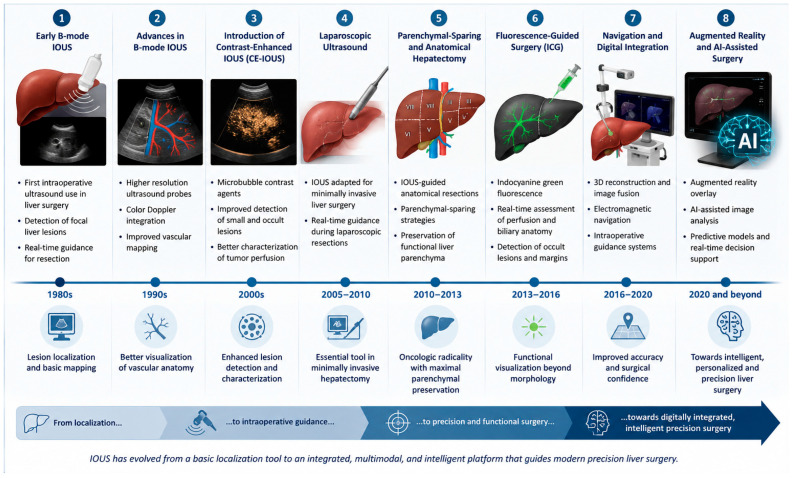
Conceptual overview of the principal intraoperative ultrasound modalities used in hepatic oncology surgery and their clinical applications. The figure highlights the major technological milestones, including advanced B-mode ultrasonography, contrast-enhanced intraoperative ultrasound (CE-IOUS), laparoscopic and robotic IOUS applications, fluorescence-guided surgery, navigation systems, augmented reality integration, and emerging artificial intelligence-assisted surgical platforms. Collectively, these developments contributed to improved lesion detection, enhanced intraoperative staging, optimization of parenchymal-sparing hepatectomy, and increased precision in hepatic oncology surgery. The ultrasound panels are schematic illustrations intended to demonstrate the characteristic imaging principles and clinical applications of each modality rather than representative patient images. Note: “This conceptual illustration was generated using ChatGPT-5 (OpenAI) based on author-designed prompts and subsequently reviewed, edited, and scientifically validated by the authors”.

**Figure 3:**
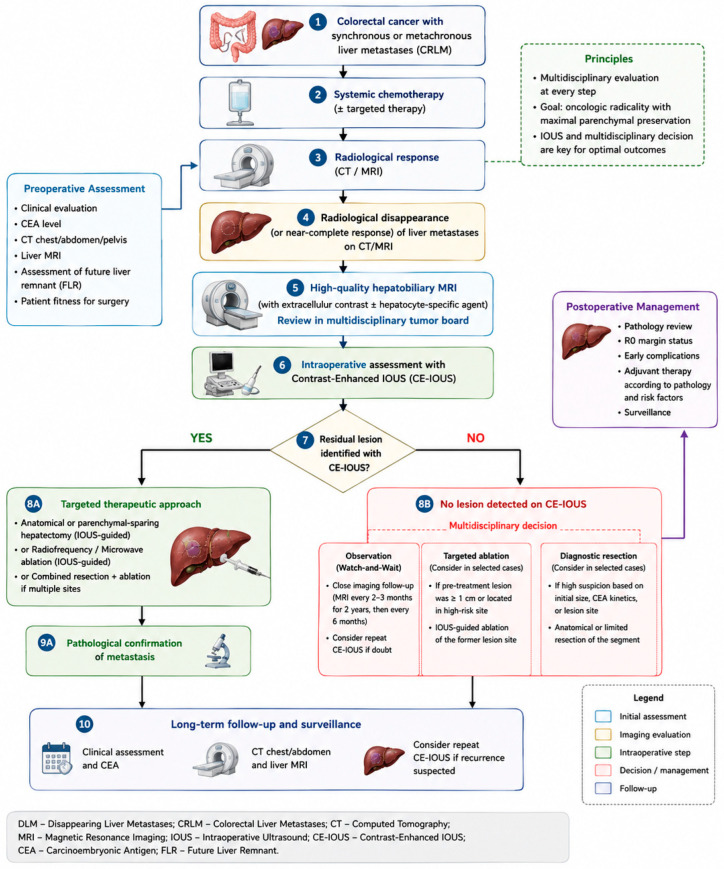
Conceptual illustration demonstrating the integration of intraoperative ultrasound (IOUS) within the modern precision-guided hepatic integrated precision surgery framework. The figure highlights the interaction between preoperative imaging, contrast-enhanced intraoperative ultrasound (CE-IOUS), fluorescence-guided surgery, laparoscopic and robotic platforms, three-dimensional reconstruction, navigation systems, augmented reality technologies, and artificial intelligence-assisted analytics. Collectively, these modalities contribute to improved lesion detection, vascular mapping, intraoperative decision-making, parenchymal preservation, and individualized oncologic liver resection. Note: “This conceptual illustration was generated using ChatGPT-5 (OpenAI) based on author-designed prompts and subsequently reviewed, edited, and scientifically validated by the authors”.

**Figure 4:**
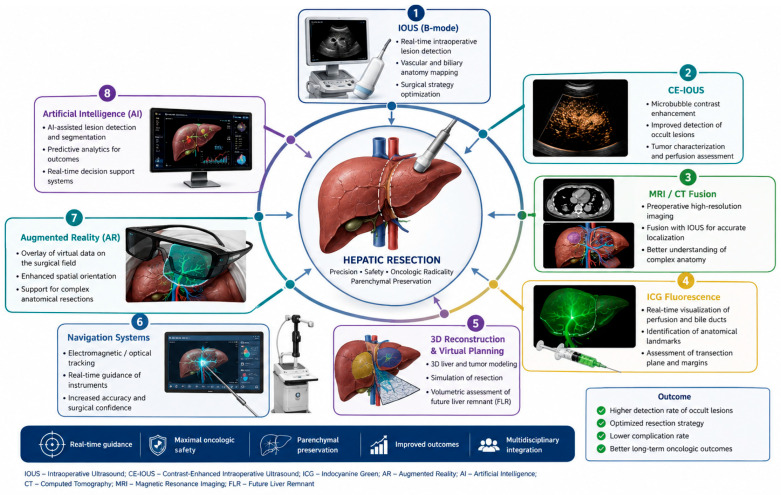
Proposed multidisciplinary management algorithm for disappearing colorectal liver metastases (DLM) following systemic chemotherapy. The figure illustrates integration of preoperative imaging reassessment, hepatocyte-specific magnetic resonance imaging, contrast-enhanced intraoperative ultrasound (CE-IOUS), and intraoperative surgical decision-making to guide lesion localization, resection strategy, ablative treatment, or postoperative surveillance. The algorithm emphasizes the central role of real-time intraoperative imaging in balancing oncologic radicality with parenchymal preservation. Note: “This conceptual illustration was generated using ChatGPT-5 (OpenAI) based on author-designed prompts and subsequently reviewed, edited, and scientifically validated by the authors”.

Figures 2–4 were developed as original conceptual scientific illustrations to facilitate visualization of complex technological and clinical concepts discussed in this review. Initial figure drafts were generated using ChatGPT-5 image-generation functionalities (OpenAI, San Francisco, CA, USA) based on author-designed prompts. The prompts were specifically developed to illustrate (1) the historical evolution of intraoperative ultrasound in hepatic oncology surgery, (2) the integration of intraoperative ultrasound within the modern precision hepatic integrated precision surgery framework, and (3) the multidisciplinary management algorithm for disappearing colorectal liver metastases. All generated images were subsequently reviewed, manually refined, scientifically validated, and approved by the authors to ensure anatomical accuracy, oncological relevance, and consistency with the published literature. The authors assume full responsibility for the scientific accuracy, validity, integrity, and final content of all figures included in this manuscript.

## 3. Results

### 3.1. Historical Evolution of Intraoperative Ultrasound in Hepatic Surgery

For the past 40 years, the use of intraoperative ultrasound (IOUS) has changed hepatic oncology surgery. Liver resection has become a precision-guided oncologic surgery and is no longer anatomy-based, as it used to be. Advances in preoperative imaging, intraoperative inspection, and manual palpation were the principal approaches, localizing lesions and determining resectability for early hepatic surgery. Despite their effectiveness, these techniques frequently underestimated the presence of tumor disease burden, occult lesions, vascular invasion, and resectable disease in patients with multifocal colorectal liver metastases or hepatocellular cancer [5,6,7,8]. Consequently, the invention of IOUS was a major leap forward in intraoperative imaging and operative planning in real time. Landmark studies have consistently shown that IOUS improves intraoperative lesion detection and frequently modifies surgical strategy by refining assessment of lesion number, localization, and tumor–vascular relationships [5,6,7,8].

In addition to its benefits for lesion detection, IOUS also affected parenchymal-sparing liver surgery. The “radical but conservative” hepatectomy concept was suggested by Torzilli and Makuuchi, who advocate the individualization of lesion resection based on IOUS information and not standardized major hepatectomy alone [11]. This idea increasingly became one of the hallmarks of modern liver surgery. Additional studies evaluating the outcomes of anatomical resections under ultrasound guidance showed that IOUS enhanced the identification of portal pedicles, hepatic veins, and segmental anatomy. This allowed customized resections that preserved liver parenchyma that was not affected by tumors in acute and chronic liver disease [12,45,46]. The introduction of CE-IOUS further expanded the diagnostic role of IOUS by enabling dynamic assessment of lesion vascularity, improving the characterization of equivocal nodules, and increasing the detection of occult or residual viable disease [13,14,15,16,17,18,19,20].

Laparoscopic ultrasound was first highlighted for its utility in the intraoperative detection of liver metastases. In their study, Russolillo et al. demonstrated that despite the modern liver MRI protocol, laparoscopic ultrasound remains useful to obtain intraoperative information during minimally invasive liver surgeries [22]. Over time, laparoscopic and robotic hepatectomy has expanded to increasingly complex liver procedures for liver cancers. Due to the increasing role of systemic chemotherapy for CRLM management, new challenges have appeared regarding disappearing liver metastases. Modern chemotherapy and biological agents have markedly increased rates of radiological complete response, although full imaging disappearance often does not equate to full pathological disappearance [23,24,25,26,47,48]. In such instances, CE-IOUS has become an important modality for the intraoperative localization of disease and parenchymal-sparing optimization. The concept of IOUS was reinforced further by the belief that it is not a diagnostic adjunct but rather an intraoperative navigation device that directly impacts surgical strategy and oncologic radicality. The increasing dissemination of ultrasound-guided parenchymal-sparing hepatectomy progressively transformed the surgical management of multifocal and bilobar hepatic disease. The landmark series by Torzilli et al. further demonstrated the clinical feasibility of this ultrasound-guided approach. Among 21 patients who underwent systematic extended right posterior sectionectomy (SERPS), both in-hospital and 90-day mortality were 0%, major postoperative morbidity occurred in only 14% of patients, blood transfusion was required in 9.5%, and no local recurrence was observed after a median follow-up of 21 months. These findings support IOUS-guided parenchymal-sparing hepatectomy as a safe oncological alternative to major hepatectomy in carefully selected patients [28].

According to Arita and colleagues, IOUS could play a crucial role in anatomical liver resection with the possibility of identifying vascular territories in real time [49]. These developments increased the indication for parenchymal-sparing liver surgery without compromising oncological safety [50,51]. Recent years have witnessed the increasing adoption of fluorescence-guided surgery, an intraoperative imaging technique that uses fluorescent contrast agents to enhance the real-time visualization of anatomical structures and tumors. The most widely used fluorophore is indocyanine green (ICG), a near-infrared fluorescent dye that accumulates in hepatic tissue and enables the visualization of liver tumors, biliary anatomy, and segmental boundaries during surgery [31,32,33]. Fluorescence imaging is particularly valuable during minimally invasive liver surgery, where it complements intraoperative ultrasound by improving identification of superficial lesions and biliary structures, while IOUS provides assessment of deeper parenchymal lesions and vascular anatomy. The use of IOUS and fluorescence-guided imaging is increasingly contributing to multimodal precision-guided hepatectomy that combines anatomical, vascular, and functional information. Recently, the development of IOUS is closely linked with various navigation systems, 3D reconstruction technologies, augmented reality platforms, and digital surgical ecosystems [52,53,54,55,56,57,58]. These innovations aim to align the information from the preoperative imaging database with real-time image information from the intraoperative ultrasound. The main goal of spatial orientation, personalized surgical planning, and intraoperative decision-making is to make it easier. Although many systems are still evolving actively, they represent an important evolution for digitally assisted and precision liver surgery overall. The future role of IOUS may be further redefined by AI and machine-learning technology.

The new AI-supported image-recognition systems have shown potential for automated lesion detection, vascular interpretation, and real-time ultrasonographic interpretation. IOUS could evolve into a comprehensive intelligent intraoperative guidance system due to its reliance on a surgical expert as well as an imaging device. Overall, the historical evolution of intraoperative ultrasonography reflects the broader transformation of hepatic surgery itself—from conventional anatomy-based resection toward individualized precision-guided oncologic intervention [59] [Table 1].

Despite major advances in preoperative imaging, IOUS continues to maintain a central role in hepatic oncology surgery because of its unique ability to provide dynamic real-time anatomical and oncologic information during operative decision-making (Figure 2).

### 3.2. Impact of Intraoperative Ultrasound on Lesion Detection and Intraoperative Staging

Accurate intraoperative staging remains one of the most important determinants of successful hepatic oncology surgery. Despite major advances in multidetector computed tomography (CT), hepatocyte-specific magnetic resonance imaging (MRI), and positron emission tomography, multiple studies continue to demonstrate that occult hepatic lesions, satellite nodules, and subtle vascular invasion may remain undetected during preoperative evaluation [5,6,7,8]. In this context, intraoperative ultrasound (IOUS) continues to play a critical role by providing dynamic real-time assessment of hepatic anatomy and tumor distribution directly during surgery.

Several early investigations demonstrated that IOUS significantly improved lesion detection compared with conventional intraoperative inspection and manual palpation alone [5,6,7,8]. Ferrero et al. reported that IOUS identified additional hepatic lesions in 18 of 225 patients (8.0%) undergoing hepatic resection for colorectal liver metastases despite contemporary preoperative CT and MRI. In three patients (1.4%), these lesions were detected exclusively by IOUS, resulting in a direct modification of the planned surgical strategy [5]. Similar findings were observed by Jrearz et al., Cohen et al., and Sietses et al., who demonstrated that intraoperative ultrasonography frequently identified additional lesions or altered surgical planning through improved characterization of lesion number, localization, and vascular proximity [6,7,8]. Overall, the available evidence suggests that the principal advantage of CE-IOUS lies not only in improved lesion detection but also in its ability to distinguish viable tumor tissue from treatment-related hepatic changes, thereby improving intraoperative decision-making. Nevertheless, most available studies are retrospective or observational and originate from specialized hepatobiliary centers, highlighting the need for larger prospective multicenter investigations to further validate these findings [13,14,15,16,17,18,19,20,21,22].

These observations established IOUS as an essential component of intraoperative staging in hepatic oncology surgery [Table 2].

It is possible to assess the relationship of the tumors with major vascular structures in a dynamic way using IOUS. Consequently, the portal pedicles, focus, proper hepatic veins, and segmental anatomy at the various transverse and coronal scanning planes are shown. This allows for the drawing up of transection planes, for each patient, to spare the uninvolved parenchyma of the liver. The use of intraoperative ultrasound allows for the continuous reassessment of static information obtained from preoperative imaging, including changing relationships during liver mobilization and parenchymal transection. Accordingly, we can alter the operative procedure depending on what we find in the course of surgery. The introduction of contrast-enhanced intraoperative ultrasound (CE-IOUS) increased the sensitivity and specificity of the staging intraoperatively [13,14,15,16,17,18,19]. Many studies suggest that CE-IOUS enhances detection of hidden hepatic lesions and persistence of viable disease in those treated by chemotherapy. We address the common issue of fibrosis and steatosis in the underlying liver, along with the challenges posed by chemotherapy-induced alterations in tissue structure on imaging. Hoareau et al. (2019) stated that CE-IOUS has enhanced detection as well as characterization of hepatic nodules in hepatic surgery at CRLM. Changes were also made to intraoperative management [18].

According to researchers, MRI before surgery was correlated with significant improvement in accurate intra-operative diagnosis during surgery with important oncological impact on HCC and CRLM. Recent studies on IOUS confirm and even enhance findings that go back decades. In one investigation, it was illustrated that IOUS is valuable even in comparison to 1.5-T MRI and MDCT imaging (i.e., imaging performed just before operation) [60]. According to Russolillo et al., the laparoscopic ultrasound still gave additional intraoperative information after a liver MRI-specific protocol was used [22]. The results of the current studies show that, as a proof-of-concept, IOUS and other designed techniques can be helpful in understanding and mapping tumors. Various studies have shown that IOUS is significant for patients with vanishing CLMs following systemic chemotherapy. A clinically complete radiological response input may not mean that complete excision of the tumor is justified. Just because there is a marked decrease in tumor size on MRI or CT, it does not mean there has been complete clearance of tumor. There might be living tumor cells located in the area even when there are no visible signs of it or evidence for it [23,24,25,26].

The use of CE-IOUS dramatically enhances intraoperative detection of hidden remaining disease, which may subsequently alter surgical and ablation tactics or monitoring decisions. IOUS can optimize both oncologic margins and vascular sparing in parenchymal-sparing hepatectomy [27,28,29], and reanalyze the trajectories of tumor resection to promote more accurate removal in composite cases. Many studies have shown the saving of major vascular structures using ultrasound-guided resections. This includes technically difficult resections involving central and large bilobar metastases [20]. According to Arita and colleagues, IOUS-assisted anatomical liver resection is useful in planning segmental/subsegmental hepatectomy for a liver tumor [43]. The evolution of minimally invasive liver surgery further consolidated the role and theory of IOUS. During laparoscopic and robotic hepatectomy, tactile feedback is not provided to the surgeon [21,22,30]. Laparoscopic ultrasound greatly relies on the surgeon for lesion location and intraoperative orientation, thus demonstrating its utility [21,22,30]. Subsequent consensus conferences on laparoscopic liver surgery were organized, with one of the basic requirements of advanced minimally invasive hepatectomy being the use of intraoperative ultrasound. Real-time intraoperative imaging guidance of minimally invasive liver surgery is a recommendation for IOUS. In spite of major advances in preoperative imaging quality, the current literature remains clearly supportive of the beneficial use of intraoperative ultrasound (IOUS) in hepatic oncology surgery. Although the available evidence consistently demonstrates that CE-IOUS improves intraoperative lesion characterization and detection compared with conventional B-mode IOUS, most studies originate from high-volume hepatobiliary centers. Consequently, although the diagnostic benefit appears convincing, broader multicenter prospective studies are still required before universal recommendations regarding its routine application can be established [13,14,15,16,17,18,19,20,21,22].

### 3.3. Contrast-Enhanced Intraoperative Ultrasound (CE-IOUS) and Characterization of Hepatic Lesions

The advent of the contrast-enhanced intraoperative ultrasound (CE-IOUS) has proven to be a great technological advance in oncologic hepatic surgery. The use of contrast-enhanced intraoperative ultrasonography (IOUS) resulted in much better diagnosis as well as lesion characterization when compared with conventional B-mode IOUS. During surgery, microbubble contrast agents could be injected to evaluate the vascular perfusion pattern on dynamic imaging. This utilization enabled a better differentiation of malignancy from benign nodules and regenerative and fibrotic chemistry-associated changes and residual viable tumor tissue. Additional studies have validated these results; the use of intraoperative ultrasonography has dramatically evolved to provide (real-time) functional oncologic imaging, instead of only anatomic guidance.

Multiple studies indicate that the detection rates for small hepatic metastases and occult lesions, often missed in the modern preoperative imaging workup, improved with the use of CE-IOUS [13,14,15,16,17]. A body of evidence suggests CE-IOUS is beneficial in surgical patients after systemic chemotherapy. In this subgroup of patients, response evaluation and chemotherapy-related alterations could be misleading on conventional imaging and diminish lesion conspicuity during intraoperative assessment. Within this context, the CE-IOUS technique enhances the visualization of vascular enhancement patterns and residual tumor perfusion with a greater sensitivity for detecting persistent metastatic disease [16,17,18,19,20]. Currently, the two most widely used ultrasound contrast agents are SonoVue^®^ (sulfur hexafluoride microbubbles), which is predominantly used in Europe, and Sonazoid^®^ (perfluorobutane microbubbles), which is primarily available in several Asian countries. Although both agents improve intraoperative lesion detection, differences in their pharmacokinetic properties and regional regulatory approval should be considered when interpreting the published literature [13,14,15,16,17,18,19,20]. Interpretation of CE-IOUS relies on dynamic evaluation of lesion enhancement patterns throughout the arterial, portal venous, and late vascular phases. Malignant hepatic lesions typically demonstrate arterial phase hyperenhancement followed by washout during the portal or late phases, whereas benign lesions generally retain contrast enhancement without significant washout. These enhancement characteristics assist the intraoperative differentiation of viable tumor from fibrosis, necrosis, or chemotherapy-related parenchymal alterations and improve lesion characterization during hepatic resection [13,14,15,16,17,18,19,20]. Although standardized CEUS LI-RADS criteria have been developed for transcutaneous liver ultrasound in patients at risk for hepatocellular carcinoma, their direct application to intraoperative CE-IOUS remains limited because intraoperative assessment is performed under different technical and clinical conditions. Consequently, CE-IOUS interpretation should always be integrated with preoperative imaging findings and intraoperative surgical assessment.

Malignant lesions of the liver typically demonstrate hyperenhancement in the arterial phase followed by washout in the portal or late phases, which enables differentiation from benign lesions as well as treatment-related parenchymal changes [13,14,15,16,17]. In contrast, chemotherapy-induced fibrosis, necrosis, and treatment-related parenchymal alterations generally demonstrate preserved or relatively homogeneous enhancement without the characteristic arterial hyperenhancement and subsequent washout pattern observed in viable malignant lesions. Recognition of these enhancement characteristics facilitates more accurate intraoperative differentiation between residual tumor and benign post-treatment changes, thereby improving surgical decision-making [18,19,20]. As a result, the presence of distorted imaging limits the observations at conventional ultrasonography. According to various authors, CE-IOUS changes intraoperative decision-making by revealing extra lesions, modifying resection planes, or causing extension of planned resections [17,18,19,20]. CE-IOUS holds significance in oncology mainly within the field of neoadjuvant chemotherapy. Hepatocyte-specific MRI and multidetector CT have substantially improved the detection of lesions preoperatively, but complete radiological disappearance does not equal complete pathological response [23,24,25,26].

Surgical specimens can show residual microscopic foci of viable tumor tissue that have disappeared radiologically on preoperative imaging, posing a high risk of undertreatment when these lesions are left unaddressed during surgery. Numerous studies have illustrated that CE-IOUS results in more accurate intraoperative localization of vanishing lesions, as well as better detection of remaining tumor tissue when compared with intraoperative imaging alone [20,23,24,25,26]. Consequently, CE-IOUS is gaining more attention.

Comparative studies evaluating CE-IOUS against contemporary MRI protocols demonstrated that the relationship between preoperative and intraoperative imaging should be considered complementary rather than competitive [22,23,24,25,26] [Table 3].

The hepatocyte-specific MRI engagement, while featuring a high level of image quality, performs well in the preoperative setting for anatomical staging and the diagnosis of small lesions. CE-IOUS offers the possibility of a reassessment at the time of mobilization and parenchymal transection in real time. Definitely, this multimodal imaging strategy may assume a role in the setting of complex hepatectomies such as those for bilobar disease, chemotherapy-induced liver damage, or lesions close to major vascular structures. CE-IOUS has a role not only in the detection of the lesions but also in the optimization of parenchymal-sparing hepatectomy. Better definition of the tumor margins and vasculature allows for tailoring the resection planes accurately while avoiding unnecessary sacrifice of functional parenchyma [27,28,29]. Numerous writers highlight how the enhancement of contrast in the body region during an ongoing operation led to better identification of the blood supply area and safe non-cancerous margin [8,11,12]. Without a doubt, these benefits may be helpful in patients with a small future liver remnant or with associated chronic liver disease. CE-IOUS has a few limitations notwithstanding its advantages. At present, the diagnostic yield continues to be somewhat operator-dependent, requiring a consistently high level of expertise in hepatic ultrasonography and the interpretation of contrast-enhancement features in relation to aspects such as imaging protocols and diagnostic criteria. The use of hepatocyte-specific contrast agent in disease of parenchymal liver, including evaluation of cirrhotic nodules, has been defined by the European and American Multimodality Working Group on Liver MRI. The present guidelines intend to homogenize the use of contrast agents in liver MRI so that diagnostic performance and accuracy can be enhanced in the characterization of cirrhotic nodules and HCC [27,28,29].

### 3.4. Disappearing Colorectal Liver Metastases After Chemotherapy

The management of disappearing colorectal liver metastases extends beyond lesion localization and directly influences decisions regarding resection, ablation, observation, and follow-up. Treating all initially documented lesions may increase operative complexity and sacrifice functional liver parenchyma, whereas leaving residual viable tumor untreated may increase the risk of local recurrence. Therefore, most contemporary strategies emphasize individualized multidisciplinary decision-making based on preoperative MRI, CE-IOUS findings, technical resectability, liver function, and oncologic risk [61,62,63,64,65].

In the modern clinical setting, the use of intraoperative ultrasound (especially contrast-enhanced) is being incorporated as a vital instrument for the intraoperative detection and assessment of disappearing CRLM. According to numerous studies, the identification of residual lesions that are undetectable on preoperative imaging can be augmented with the utilization of CE-IOUS. The observation of dynamic vascular enhancement alteration patterns associated with residual tumor may guide the intraoperative diagnosis of subtle residual tumor perfusion within fibrotic, chemotherapy-influenced liver parenchyma. Enhancing intraoperative staging accuracy and diminishing the premature treatment risks from misconstruing preoperative imaging involves adopting this approach. Hepatocyte-specific magnetic resonance imaging, which is used preoperatively to identify disappearing lesions, helps improve the identification of these lesions dramatically; however, several studies continue to advocate the role of intraoperative ultrasonographic guidance [22,23,24,25,26].

In 2019, Owen et al. established that the use of hepatobiliary contrast-enhanced MRI aided in the detection of lesions but did not affect the requirement for careful intraoperative assessment [26]. Taken together, the current evidence suggests that disappearing colorectal liver metastases should not automatically be considered completely eradicated. Oba et al. demonstrated that, despite the introduction of hepatocyte-specific MRI and CE-IOUS, residual viable tumor remained detectable in a proportion of disappearing colorectal liver metastases. Their findings emphasize that radiological disappearance should not be interpreted as definitive pathological complete response and support continued intraoperative assessment using CE-IOUS to guide surgical management [25]. Therefore, individualized multidisciplinary decision-making remains the most appropriate strategy until higher-level prospective evidence becomes available [22,23,24,25,26,27,28,29,61,62,63,64,65].

Consequently, modern management strategies increasingly rely on combined preoperative MRI and intraoperative ultrasonographic assessment rather than considering either modality to be independently sufficient (Figure 3).

The clinical implications of disappearing colorectal liver metastases extend well beyond lesion localization, influencing surgical planning, the extent of liver resection, and long-term oncological management. As far as surgical resection or ablation admission, observation or prolonged neoadjuvant chemotherapy, or extensive parenchymal sacrifice is concerned, there is considerable controversy. The upfront treatment of every lesion recognized will increase operative complexity and needless sacrifice of functioning liver tissue. By contrast, neglecting to address areas with leftover tumor foci may lead to local recurrence and poor oncologic outcome. As a result, numerous researchers recommended that systematic therapy be administered to all initially identified metastatic sites as far as technically doable, especially in patients at low operative risk [62,63,64,65].

In addition, the evolving philosophy that favors parenchymal sparing for hepatectomy complicated the management of disappearing lesions. Over the years, the goal of contemporary liver surgery has been to preserve functional liver reserve as much as possible. This is essential in the case of patients who have bilobar disease, liver injury from chemotherapy, and those with a possibility of future recurrence [27,28,29]. In this context, correct intraoperative localization was essential to balancing oncological radicality with preservation of uninvolved parenchyma. As a result, CE-IOUS-guided targeted resections and ultrasound-guided ablation strategies are likely to offer an optimal compromise between adequate oncologic treatment and unnecessary hepatic sacrifice. Managing disappearing colorectal liver metastases (CRLM) varies greatly between hepatobiliary centers. There is substantial variation in the criteria for resection and follow-up.

Clinics often recommend the resection of all lesions initially documented, regardless of the radiological response, after the first two cycles. Other centers recommend selective surveillance approaches for patients who exhibit durable radiological complete response following chemotherapy. There is no standard protocol due to variation in outcomes, as shown above. Moreover, the biological nature of vanishing lesions is still not clearly defined. Recent research underlines the importance of multidisciplinary decision-making in patients with vanishing CRLM.

To make management feasible, integrated oncologic response patterns, MRI findings, intraoperative ultrasonographic findings, technical resectability, liver function, future treatment options, and plans must be integrated. Consequently, disappearing metastases are increasingly a paradigm of precision oncologic surgery, which enables intraoperative imaging and dynamic integration of systemic therapy response and personalized surgical planning. The conclusion is that the available evidence for disappearing colorectal liver metastases continues to support the value of IOUS and CE-IOUS. Despite advancements in MRI and chemotherapy, intraoperative ultrasound techniques are significant with respect to disappearing lesions and other liver lesions. These may help in accurately localizing the tumors, optimizing parenchymal-sparing strategies, and maintaining oncologic radicality during hepatic resection.

### 3.5. Intraoperative Ultrasound and Parenchymal-Sparing Hepatectomy

The development of liver surgery is one of the most important conceptual advances in hepatobiliary oncology. Indeed, major anatomical hepatectomies have almost always been replaced by this more conservative parenchymal-sparing hepatectomy as the standard operation for colorectal liver metastases and hepatocellular carcinoma [27,28,29]. Wider anatomical resections were associated with better oncologic radicality in the past. Under a simple surgical philosophy, patients were subjected to major anatomical hepatectomies. A developing knowledge of liver regeneration, post-hepatectomy liver failure, repeat hepatectomy, and long-term oncologic management gradually changed this philosophy [27,28,29]. As a result, the liver surgery principle of preserving functional liver tissue was born. Thus, all liver surgery types try to spare uninvolved hepatic tissue whenever technically and oncologically feasible. Intraoperative ultrasound has been instrumental in performing parenchymal-sparing liver surgery by providing information on the intra-liver topography of tumor nodules [27,28,29,34].

The IOUS system makes it possible to visualize various anatomical characteristics in real time in the operating room. Surgical guidelines, which rely solely on preoperative imaging, are inflexible, restricted to the opening stage of the operation, and are not dynamic. Torzilli et al. developed an IOUS-guided liver resection based on intraoperative vascular mapping and individualized transection planes rather than anatomical resections [27,28]. The technical characteristics of these resections enabled preservation of the main vascular structures and hepatic segments in patients with multiple bilobar metastases or lesions close to the main vascular pedicles. A growing body of literature indicates that parenchymal-sparing surgery under intraoperative ultrasound (IOUS) guidance is associated with lower rates of postoperative liver failure and better liver remnant preservation, and enhanced repeat hepatectomy rates for recurrent disease [52,65,72,73]. The most important benefit of parenchymal-sparing hepatectomy is the ability to maintain hepatic functional reserve postoperatively. This could be especially relevant in patients with chemotherapy-induced liver damage, steatosis, cirrhosis, chronic liver disease, or multiple CRLM with planned serial resections [34,53]. Indeed, the use of IOUS to guide parenchymal-sparing liver resection has the potential to reduce postoperative morbidity by preventing the unnecessary resection of uninvolved parenchyma and retaining the option of salvage surgery in case of recurrence. Several studies reported on the significant contribution of repeat interventions on the liver to long-term survival in selected oncological patients [27,28,29].

Furthermore, IOUS purposefully enables the improved identification of intraoperative lesions, enabling safer dissection, and the selective vascular-sparing parenchymal transection is a real-time visualization of hepatic veins, Glissonian pedicles, portal bifurcations, and venous drainage pattern [27,28,29,74]. As this concerns tumors situated adjacent to complementary vascular structures, the current standards would have necessitated responsive major hepatectomy. Recent IOUS-guided techniques are allowing more limited anatomical and non-anatomical resections with adequate surgical margins and vascular sparing. The CE-IOUS further improved parenchymal-sparing surgery results via enhancement of delineation and detection of occult satellite nodules [13,14,15,16,17]. The intraoperative appraisal of the true extent of the neoplasm may be aided by CE-IOUS, which may permit more accurate resection plane tailored to tumor extent. Liver parenchyma that has undergone chemotherapy may exhibit fibrosis and treatment-related changes. Such changes can potentially affect the sonographic interpretation intraoperatively. The above will minimize the chance of positive surgical margins and additionally avoid an unwarranted increase in hepatic resection. Parenchymal-sparing hepatectomy has recently gained significance in minimally-invasive liver surgery. Liver surgery by laparoscopy and robotics favors limited resections because of technical considerations on exposure, vascular control, and preservation of liver function [30,38,39,40,41]. Laparoscopic ultrasound is an essential intraoperative navigation modality to locate lesions and determine transection planes in these cases.

IOUS can support as well as guide localized or anatomic subsegmental resections and support precision targeting of these lesions [37]. It can efficiently localize “missing” tumors requiring more extensive resection, which would otherwise compromise liver function [38]. Several studies showed that IOUS-guided minimally invasive resections can achieve oncologic results similar to open surgery while still maintaining the benefits of lowered surgical trauma and faster postoperative recovery [38,39,40,41,42,43]. For all its merits, parenchymal-sparing surgery still remains technically demanding. It is also still extremely operator-dependent and requires expertise in intraoperative ultrasonography. The accurate interpretation of vascular anatomy, the assessment of functional liver territories, the decisions regarding the extent of resection, and the real-time modification of the surgical strategy require extensive previous experience in hepatobiliary surgery and advanced IOUS techniques. Learning curve effects and institutional expertise biases are still present in the outcome literature. Although parenchymal-sparing hepatectomy has become increasingly accepted, its successful implementation remains highly dependent on meticulous IOUS guidance and surgeon experience. Current evidence suggests that, when appropriately selected, this approach can preserve functional liver parenchyma without compromising oncological outcomes; however, careful patient selection remains essential [52,53,54,55,56,57,58,73].

### 3.6. Role of Intraoperative Ultrasound in Laparoscopic and Robotic Liver Surgery

The rapid development of minimally invasive liver surgery has enabled the strong and abundant integration of intraoperative ultrasound (IOUS) as a guiding device for hepatic resection [21,22,30]. In laparoscopic and robotic surgery, direct palpation of the tumor is indeed not possible, and tactile feedback is decreased in robotic surgery, which makes intraoperative real-time imaging crucial for lesion detection, orientation and visualization of vessels, guidance, and evaluation of safe transection lines [21,22,30]. The role of laparoscopic intraoperative ultrasound is evolving with the passage of time, expanding from an adjunct device designed to provide additional imaging information to a vital component of minimally invasive surgery for hepatic oncologic surgery [21,22]. According to early studies based on laparoscopic ultrasonography, this examination has a significant role in finding lesions [21]. Hartley and colleagues were the first to discuss the laparoscopic use of ultrasound for colorectal surgery for liver metastases, demonstrating that laparoscopic intraoperative ultrasound is a crucial device to compensate for the absence of liver palpation [21].

Many subsequent analyses revealed the presence of extra lesions with laparoscopic IOUS, which changes operative strategy a lot of the time despite laborious best preoperative MRI protocols [22]. The utility of laparoscopic ultrasound for staging patients with colorectal liver metastases is still high, as shown by Russolillo et al., in comparison with the best liver-specific MRI protocol and liver-specific contrast agents [22].

This technique’s key benefit is real-time visualization of the vascular anatomy and tumor-to-vessel relationship during transection of the parenchyma. Such a technique further allows for dynamic evaluation of the portal pedicles, hepatic veins, and segmental borders, allowing tailoring of the transection plane and performing of parenchymal-sparing hepatectomy according to individual anatomy and location of tumors. A laparoscopic anatomical resection can be performed using a parenchymal-sparing approach [27,28,29,30]. Afterward, segmental or subsegmental pedicle dissection is conducted under real-time sonographic source imaging, utilizing the proximity assessment of the tumor to the vessels. This is performed to enable en-bloc or parenchymal-sparing resection. The significant roles played by IOUS, the ability to visualize and localize vessels, offer essential support during posterior segment resections, centrally located tumors, and multifocal bilobar diseases. The optimization of laparoscopy can be difficult due to anatomical orientation; however, IOUS makes it simpler. Laparoscopic parenchymal-sparing hepatectomy is becoming increasingly popular [31,32,33,34,35,36,37].

As a result, laparoscopy-guided IOUS is increasingly employed for surgical planning and execution, thus becoming a strategic rather than a complementary factor during minimally invasive resections. Many investigations revealed that minimally invasive ultrasound-guided resections yield similar oncological results compared to open surgery. This offers advantages related to smaller operative trauma, less time in hospital, reduced postoperative pain, and quicker recovery. The Southampton Consensus Guidelines also emphasized the important role of IOUS in lesion localization, vascular anatomic mapping, and intraoperative orientation during minimally invasive liver surgery [40]. These guidelines reflected a developing consensus that reliable intraoperative ultrasonographic guidance is necessary if complex laparoscopic liver surgery is to be safely performed. Robotic liver surgery further reinforces the need for intraoperative imaging, because tactile feedback is limited or absent. In this setting, IOUS remains essential for lesion localization, anatomical orientation, and guidance during both minor and major robotic hepatectomies [66,67,68,75,76,77].

Similarly, there is still a requirement for imaging during the procedure. Indeed, the absence of touch feedback during robotic surgery might even heighten dependence on IOUS for lesion localization and anatomical orientation during minimally invasive hepatectomy. Positive outcomes were reported with robot-assisted liver surgery; however, this signifies that accurate intraoperative guidance is paramount during minor or major hepatectomy [66]. In their research, Giulianotti et al. presented the utilization of intraoperative imaging during totally robotic right hepatectomy [67]. Fluorescence-guided surgery is another new concept benefiting image-guided minimally invasive hepatectomy development. Fluorescence imaging has been made possible through a dye called indocyanine green (ICG).

Combining fluorescence imaging and intraoperative ultrasound imaging (IOUS) is becoming an increasingly common multimodal intraoperative imaging strategy. Ultrasonography provides important information on deep parenchyma and vasculature. Conversely, fluorescence imaging is useful for improving the identification of superficial correlation for anatomical orientation. Their combined use may be especially useful during laparoscopic and robotic resections where spatial orientation remains technically difficult. The advancement of navigation systems and digitally assisted operative platforms was also expedited by ultra-minimally invasive liver surgery [52,53,54,55,56,57,58]. The accuracy of intraoperative orientation during complex resections may be improved with the employment of real-time integration of laparoscopic ultrasound with 3D reconstruction technologies, virtual sonography, and augmented reality systems. Satou et al. proposed that real-time virtual sonography can be used for intraoperative navigation during liver resection. This indicates a merging tendency of ultrasonography and computer-assisted surgery technologies [54]. IOUS is increasingly at the forefront of precision hepatic surgery that is digitally integrated. IOUS is less invasive but has significant technical limitations despite considerable technological developments. Accurately interpreting ultrasound during laparoscopy requires significant hepatobiliary-related knowledge, orientation/understanding of the laparoscopic view, and image acquisition. Learning curve effects continue to be important, especially for major hepatectomy, posterior segment resections, and anatomically complex procedures. According to Brown and Geller, laparoscopic major hepatectomy has a steep learning curve, and Saito et al. suggest structured training for minimally invasive liver surgery and standardized procedures [78,79].

Nevertheless, the cumulative evidence strongly supports the indispensable role of IOUS in modern laparoscopic and robotic hepatic oncology surgery. As minimally invasive techniques continue to expand toward increasingly complex resections, intraoperative ultrasonography remains essential for lesion localization, vascular mapping, parenchymal preservation, and maintenance of oncologic radicality. Contemporary minimally invasive liver surgery therefore increasingly depends on multimodal image-guided operative strategies centered around real-time intraoperative ultrasonographic guidance (Figure 4).

### 3.7. Navigation Systems, Augmented Reality, and Digital Liver Surgery

With the increasing complexity of oncology surgery, navigation systems and digitally-assist platform have been developed to improve intraoperative orientation, as well as oncologic accuracy during resection. Although intraoperative ultrasound (IOUS) is still the gold standard for real-time intraoperative imaging, ongoing technological innovations have recently resulted in the combination of ultrasonographic guidance with three-dimensional reconstruction, virtual simulation, augmented reality (AR), electromagnetic tracking systems, and computer-assisted navigation systems. On the whole, such platforms facilitated the commencement of digitally assisted precision liver surgery. The major limitation of standard hepatic surgery is the discrepancy between static preoperative images and dynamic intraoperative anatomy. During surgery, the anatomical relationship may be altered due to respiratory motion, liver mobilization, deformation of the parenchyma, displacement of vessels, and manipulation, thereby restricting their direct application [52,53,54,55,56,57,58]. As a result, they associate preoperative radiological sets and intra-operative image systems to assist in spatial orientation and personalized surgical planning. Research has demonstrated that the use of image-guided navigation platforms may significantly improve the intraoperative localization of hepatic lesions [52,53,54,55,56,57,58]. They are also able to enhance understanding of complex vascular anatomy. Researchers investigated the feasibility of live virtual sonography in intraoperative navigation during liver resection [54].

To enhance anatomical references, the preoperative imaging dataset and intraoperative ultrasonography are combined. According to Oldhafer et al., navigation systems are currently one of the most important future challenges of hepatobiliary surgical research, as they provide essential support for transforming hepatic surgery into a digitally assisted discipline [56]. Another advance of digitally assisted surgery of the liver is 3D reconstruction. The authors mention that such a platform can enable the preoperative simulation of liver resection [80]. You can now preoperatively assess the relationship of the tumor to the vessels, segmental anatomy, future liver remnant volume, and predetermined transection planes. Three-dimensional simulation software is valuable to the planning of anatomical segmentectomy and subsegmentectomy [81]. This is particularly applicable to patients who have vascular accessory or derived anatomy, or central tumors. Theoretically, the combined use of IOUS with these technologies could enhance the execution of personalized strategies by improving intraoperative anatomy identification. Electromagnetic tracking systems and real-time image fusion technologies represent other relevant developments in intraoperative navigation. According to Ivashchenko et al., these frameworks are designed to counter intraoperative liver distortion and allow greater precision synchrony of preoperative imaging with operative anatomy. These tools may prove useful during less invasive surgical procedures and parenchyma-saving resections that demand precise spatial orientation [57].

Navigating resection may provide clinical benefits in relation to the discovery of disappearing colorectal liver metastases. Olthof and colleagues developed a method for identifying and rectifying the disappearance of advanced colorectal liver metastases by combining historical imaging data with intraoperative ultrasonographic navigation guidance [66]. These systems may help identify metastatic sites that were previously identified but which became occult due to chemotherapy. This may allow oncologic radicality without the unnecessary sacrifice of healthy liver parenchyma.

According to recent software developments, augmented reality (AR) will help in the conceptualization of digital liver surgery. The application of AR will help in directly superimposing the reconstructed anatomical information of the liver and hepatic tumor on either the surgical site or the display of laparoscopy [70,71]. Different AR platforms aim to merge preoperative imaging, intraoperative ultrasound, and vascular anatomy with the planned transection planes into a single visual space that enhances operative orientation and anatomical understanding intuitively. According to recent work by Oh and co-workers, augmented reality-guided three-dimensional laparoscopic liver surgery is feasible, indicating an increasing incorporation of digital visualization technologies into minimally invasive surgical hepatic oncology [82]. Oh and colleagues presented a survey on digital intelligent liver surgery. This addresses digital and intelligent liver surgery that goes beyond navigation, instead opting for multimodal integration of imaging, AI, surgical simulation, and real-time intraoperative guidance [82]. According to Fang et al., digital liver surgery is a new paradigm that can be defined as the use of advanced computational tools allowing individualized operative planning, enhanced intraoperative accuracy, and thus more and more personalized oncologic therapies [69]. Under this paradigm, IOUS continues to serve as the primary real-time imaging interface that connects navigation systems, virtual reconstructions, and augmented reality. There are technological advances, but their implementation and diffusion in navigation-assisted liver surgeries is greatly constrained. Errors related to respiratory motion, liver deformation, and intraoperative mobilization are not registered.

Because ultrasonography is increasingly being used in a three-dimensional manner for the planning and guidance of resection, the acceptance of virtual three-dimensional simulation for preoperative planning and intraoperative guidance is increasing. Nevertheless, numerous navigation systems continue to rely on institutions, demand advanced technologies, and incur higher infrastructural costs. The impact of learning curve effects remains significant, especially with the mixing of ultrasonographic reading with virtual navigation systems and augmented reality systems. Liver surgery is increasingly evolving from conventional anatomy-based resection toward integrated image-guided precision surgery where intraoperative ultrasound plays a central role.

### 3.8. Artificial Intelligence and Future Perspectives in Precision Hepatic Surgery

Artificial intelligence (AI), machine learning, and advanced computational imaging technologies are increasingly redefining the future of hepatic oncology surgery. Although intraoperative ultrasound (IOUS) remains fundamentally dependent on surgeon expertise and real-time interpretation, modern digital technologies increasingly aim to augment intraoperative decision-making through automated image analysis, intelligent navigation systems, and AI-assisted surgical guidance [69,70,71]. These developments suggest that hepatic surgery is progressively transitioning toward a precision-integrated, precision-guided surgical platform in which intraoperative imaging, computational analysis, and digital navigation function synergistically to improve oncologic and technical outcomes.

One of the principal limitations of conventional IOUS remains its operator dependency. Accurate interpretation of ultrasonographic findings requires substantial expertise in hepatobiliary anatomy, vascular mapping, lesion characterization, and contrast-enhanced intraoperative ultrasound (CE-IOUS) analysis [13,14,15,16,17,18,19,20]. Variability in surgical experience may therefore influence lesion detection rates, intraoperative decision-making, and oncologic adequacy. AI-assisted image-processing systems have consequently emerged as promising tools for improving standardization and reducing interpretation variability during hepatic surgery [69,70,71].

Recent investigations evaluating AI-assisted ultrasonographic analysis demonstrated encouraging results regarding automated lesion recognition and the characterization of hepatic tumors. Machine-learning algorithms trained using large imaging datasets may facilitate the identification of subtle metastatic deposits, differentiate treatment-related fibrosis from residual viable tumor, and assist the interpretation of vascular enhancement patterns during CE-IOUS [69,70,71]. These technologies may become particularly valuable in chemotherapy-treated livers where parenchymal alterations frequently complicate conventional ultrasonographic interpretation.

Artificial intelligence also has the potential to significantly improve preoperative planning and surgical simulation. Digital platforms integrating three-dimensional reconstruction, volumetric analysis, vascular segmentation, and predictive modeling may facilitate individualized selection of resection strategies and optimization of future liver remnant preservation [71,80]. In this context, AI-assisted systems may support increasingly personalized operative planning based on patient-specific oncologic and anatomical characteristics. Such approaches may be particularly relevant in patients with multifocal colorectal liver metastases, centrally located tumors, or complex vascular anatomy requiring highly individualized resection strategies.

The integration of AI with navigation systems and augmented reality platforms further expands the concept of intelligent liver surgery [70]. Real-time synchronization between preoperative imaging, intraoperative ultrasound, and computer-assisted visualization technologies may improve spatial orientation during hepatic resection and facilitate intuitive interpretation of complex anatomical relationships. Augmented reality-assisted surgery may ultimately permit dynamic projection of vascular structures, tumor boundaries, and planned transection planes directly onto the operative field, thereby enhancing surgical precision and intraoperative confidence.

Minimally invasive liver surgery represents a particularly suitable environment for AI-assisted technologies because laparoscopic and robotic procedures already rely heavily on video-based operative platforms [30,66,67,68]. AI-assisted image registration, automated anatomical recognition, and real-time intraoperative segmentation may improve orientation during laparoscopic and robotic hepatectomy, especially in anatomically complex resections involving deep parenchymal lesions or posterior hepatic segments. In robotic surgery, where tactile feedback is absent, intelligent image-guided systems may further compensate through enhanced digital visualization and automated anatomical assistance.

Emerging technologies also aim to improve the intraoperative prediction of oncologic margins and vascular preservation. Deep-learning algorithms capable of integrating radiological, intraoperative, and pathological data may potentially assist the prediction of tumor infiltration patterns and optimization of parenchymal-sparing strategies [69,70,71]. Such developments could eventually support more individualized balancing between oncologic radicality and preservation of functional liver reserve.

Despite substantial technological enthusiasm, several important limitations continue to restrict clinical implementation of AI-assisted hepatic surgery. Most currently available studies remain experimental, retrospective, or proof-of-concept investigations performed in highly specialized centers [69,70,71]. Standardization of imaging datasets, validation of machine-learning algorithms, integration into operative workflows, and real-time processing capabilities remain incompletely established. Furthermore, AI-assisted systems remain highly dependent on image quality, computational infrastructure, and operator interaction.

Recent studies suggest that artificial intelligence-assisted image analysis has the potential to improve intraoperative lesion detection, vascular identification, and surgical decision support. However, the currently available evidence remains preliminary, and further prospective clinical validation is required before routine implementation in hepatobiliary surgery [70,71].

Additional ethical and practical considerations must also be acknowledged. Increasing integration of artificial intelligence into surgical decision-making raises important questions regarding algorithm transparency, medico-legal responsibility, cybersecurity, data governance, and preservation of surgeon autonomy. Consequently, current evidence supports the role of AI as an adjunctive decision support technology rather than a replacement for surgical expertise and intraoperative judgment. In this evolving framework, IOUS is likely to remain the central real-time imaging interface linking preoperative imaging, navigation systems, augmented reality, and AI-assisted decision support.

### 3.9. Current Limitations and Emerging Ultrasound Technologies

Despite its well-established role in hepatic oncology surgery, intraoperative ultrasound (IOUS) has several important limitations that continue to influence its widespread implementation and clinical reproducibility. One of its principal limitations remains its marked operator dependency. Accurate intraoperative interpretation requires extensive expertise in hepatobiliary anatomy, ultrasonographic image acquisition, vascular mapping, lesion characterization, and real-time surgical decision-making. Consequently, the diagnostic accuracy and intraoperative utility of IOUS may vary considerably according to the surgeon’s experience and institutional expertise. The learning curve for advanced IOUS remains substantial, particularly during complex anatomical liver resections, minimally invasive procedures, and the management of multifocal or centrally located hepatic tumors [52,74,83,84,85,86]. Technical limitations also deserve consideration. Image quality may be affected by liver cirrhosis, steatosis, chemotherapy-associated liver injury, fibrosis, or altered tissue echogenicity, potentially reducing lesion conspicuity and complicating the differentiation between viable tumor tissue and treatment-related parenchymal changes [16,17,18,19,23,24,25,26,27,28,29,52]. Furthermore, conventional IOUS remains limited by acoustic artifacts, restricted acoustic windows, and the inability to directly identify microscopic tumor infiltration. Variability in imaging protocols and the absence of standardized interpretation criteria may also contribute to interobserver variability and differences in surgical decision-making across hepatobiliary centers [52,56,74,83,84,85,86]. Future developments are expected to address several of these limitations through the integration of artificial intelligence-assisted image analysis, three-dimensional reconstruction, multimodal image fusion, navigation systems, and augmented reality platforms. These technologies may improve intraoperative image interpretation, facilitate anatomical orientation, reduce operator dependency, and support increasingly standardized and precision-guided hepatic resections [54,56,57,58,69,70,71,78,79,80,81,82,87].

In parallel, ongoing advances in ultrasound technology continue to expand the potential capabilities of intraoperative imaging. Emerging techniques, including microvascular (microflow) ultrasound and super-resolution ultrasound imaging (ultrasound localization microscopy), are being investigated as novel methods for improving visualization of tissue vascularity and microvascular architecture. Although their application in hepatic oncology surgery remains at an early stage, these technologies illustrate the continuing evolution of ultrasound imaging and may further complement conventional and contrast-enhanced intraoperative ultrasound as experience and clinical evidence continue to grow [52,56,57,58,69,70,71,78,79,80,81,82,87].

Overall, artificial intelligence should currently be regarded as an adjunct rather than a replacement for surgical expertise. Although early studies demonstrate promising improvements in image interpretation and intraoperative guidance, robust prospective validation and external multicenter evaluation remain necessary before widespread clinical implementation [69,70,71,78,79,80,81,82,87,88].

## 4. Discussion

The present structured narrative review underscores the continuing fundamental role of intraoperative ultrasound (IOUS) in contemporary liver oncology surgery, despite the major advances in preoperative imaging. The most recent literature indicates that hepatic imaging using high-resolution magnetic resonance imaging (MRI), multidetector computed tomography (CT), and hepatocyte-specific contrast agents has made huge strides in matching the accuracy of preoperative staging. Despite this confidence, the intraoperative ultrasound (IOUS) has proved to be essential. Indeed, IOUS remains significant for lesion detection, vascular mapping, intraoperative staging, and personalized surgical planning in real time. As the literature shows, IOUS still has significant detection value for occult lesions and operative strategy change. According to these results, liver surgery fundamentally depends on the dynamic intraoperative anatomical evaluation rather than the static radiological evaluation. Within this framework, IOUS becomes not just a diagnostic adjunct, but an intraoperative real-time navigation that influences oncologic decisions. The oncologic utility of intraoperative imaging was further expanded by CE-IOUS development.

The assessment of data shows CE-IOUS enhances the characterization of liver lesions and the detection of viable fibrotic lesions, especially in the setting of chemotherapy-treated liver or disappearing colorectal liver metastases (DLM) [13,14,15,16,17,18,19,20,21,22,23,24,25,26]. As we delve into this subject, we are in the era of modern systemic therapy in which radiological complete response is becoming increasingly common, but incomplete pathological response is still the case. The administration of DLM probably appears to be the most obvious proof of the constant ongoing requirement for very careful and extensive intra-operative ultrasound evaluation during liver resection. A topic that arises often in the literature is the gradual transition to parenchymal-sparing hepatectomy with the assistance of IOUS [27,28,29]. Due to extended long-term survival, repeat hepatectomy feasibility, and the increasing rate of liver damage related to chemotherapy, the preservation of functional hepatic reserve has gained increasing importance. The vascular-oriented resection plan guides the surgeon and provides excellent visualization to properly resect the area without damaging neighboring tissues. The increased use of laparoscopic and robotic liver surgery has increased the strategic value of intraoperative ultrasound in recent years [30,38,39,40,41,42,43,66,67,68].

Emerging tools for interoperative liver lesion imaging are digital/hybrid operating theater platforms that facilitate the integration of laparoscopic ultrasound video images and external computed tomography imaging of the liver, as well as novel robotic laparoscopic ultrasound probe control systems. As per the history of liver surgery, new operative techniques and approaches are likely to evolve in the near future, as there have been changes in vascular and biliary resection techniques. Thus, the demand for advanced image-guided surgery solutions will further expand. More novel operative concepts, such as vascular inflow/outflow modification through preoperative radiological portal and hepatic vein embolization, as well as more aggressive procedures such as staged vascular resection, two-stage hepatectomy, and associating liver partition and portal vein ligation for staged hepatectomy, will probably be associated with more complex ultrasound imaging during surgery. In addition to its well-established clinical applications, it is important to acknowledge that IOUS remains associated with several limitations, including operator dependency, the need for specialized training, and variability in image interpretation. The integration of emerging technologies, such as artificial intelligence-assisted image analysis, multimodal image fusion, navigation systems, and advanced ultrasound imaging techniques, has the potential to address many of these challenges by improving image interpretation, enhancing intraoperative guidance, and reducing interobserver variability. As these technologies continue to evolve, they are expected to further strengthen the role of IOUS within precision-guided hepatic oncology surgery while promoting greater standardization and reproducibility of intraoperative decision-making.

Although the current literature consistently supports the clinical utility of IOUS, much of the available evidence is derived from retrospective cohort studies, single-center experiences, and expert consensus statements. Consequently, differences in patient selection, institutional expertise, ultrasound equipment, and operative strategies may limit the generalizability of published results. Future multicenter prospective studies and standardized reporting are warranted to further define the role of IOUS across different hepatobiliary surgical settings. The strongest clinical evidence supporting IOUS has been generated in hepatocellular carcinoma and colorectal liver metastases, and its application continues to expand to other primary and secondary hepatic malignancies. In particular, IOUS may facilitate intraoperative staging, vascular mapping, and margin assessment in patients with intrahepatic cholangiocarcinoma and selected non-colorectal liver metastases. Nevertheless, prospective studies evaluating these indications remain limited, and further research is required to better define the clinical benefits of IOUS in these patient populations [52,69,70,71,74,78,79,80,81,82,83,84,85,86,87,88,89].

Overall, the available evidence supports IOUS as a central real-time imaging modality in precision hepatic surgery. Its integration with CE-IOUS, minimally invasive surgery, fluorescence imaging, navigation systems, and AI-assisted technologies may further improve intraoperative accuracy, standardization, and oncologic safety.

## 5. Conclusions

Intraoperative ultrasound remains a fundamental component of modern hepatic oncology surgery despite major advances in preoperative imaging technologies. The available evidence demonstrates that IOUS and contrast-enhanced intraoperative ultrasound significantly improve lesion detection, intraoperative staging, vascular mapping, and precision-guided surgical planning, particularly in patients with colorectal liver metastases and hepatocellular carcinoma. Their role is especially relevant in the management of disappearing colorectal liver metastases and during parenchymal-sparing hepatectomy, where accurate real-time intraoperative assessment directly influences oncologic radicality and the preservation of functional liver reserve.

The continued expansion of laparoscopic and robotic liver surgery has further reinforced the importance of intraoperative ultrasonographic guidance, while emerging navigation systems, augmented reality platforms, and artificial intelligence technologies are progressively transforming hepatic surgery toward digitally assisted precision oncology. Future developments will likely integrate IOUS into increasingly intelligent multimodal surgical ecosystems, maintaining its central role in the evolution of individualized image-guided hepatic surgery.

## Figures and Tables

**Table 1:** Major clinical applications of intraoperative ultrasound in hepatic oncology surgery.

Clinical Application	Role of IOUS	Main Surgical Benefit	Key References
Detection of occult hepatic lesions	Identification of small metastases and satellite nodules not detected preoperatively	Improved intraoperative staging and oncologic assessment	[5,6,7,8,60]
Contrast-enhanced lesion characterization (CE-IOUS)	Dynamic evaluation of vascular perfusion and residual viable tumor	Improved differentiation between malignant lesions, fibrosis, and chemotherapy-related changes	[13,14,15,16,17,18,19,20]
Intraoperative vascular mapping	Real-time visualization of portal pedicles and hepatic veins	Safer anatomical liver resections and preservation of vascular integrity	[27,28,29]
Parenchymal-sparing hepatectomy	Guidance of individualized transection planes	Preservation of functional liver reserve and reduction of postoperative liver insufficiency	[27,28,29,30,31,32,33,34]
Management of disappearing CRLM	Localization of occult residual metastatic disease after chemotherapy	Optimization of oncologic radicality while minimizing unnecessary liver sacrifice	[23,24,25,26,61,62,63,64,65]
Laparoscopic liver surgery	Real-time lesion localization in the absence of tactile feedback	Improved minimally invasive surgical navigation and operative precision	[21,22,38,39,40,41]
Robotic liver surgery	Intraoperative orientation and anatomical guidance	Enhanced precision during robotic hepatic resections	[66,67,68]
Navigation-assisted hepatic surgery	Integration with 3D reconstruction and augmented reality platforms	Improved spatial orientation during complex resections	[52,53,54,55,56,57,58]
Fluorescence-guided precision surgery	Combination with indocyanine green imaging and multimodal visualization	Enhanced tumor and biliary anatomy identification	[31,32,33]
AI-assisted hepatic surgery	Automated image analysis and intelligent intraoperative guidance	Potential future optimization of precision-guided liver surgery	[69,70,71]

Major contemporary clinical applications of intraoperative ultrasound in hepatic oncology surgery summarized from the hepatobiliary surgical oncology literature, emphasizing its role in lesion detection, intraoperative staging, parenchymal-sparing hepatectomy, minimally invasive liver surgery, and digitally assisted precision hepatic surgery.

**Table 2:** Landmark studies evaluating IOUS and CE-IOUS in hepatic oncology surgery.

Author	Year	Study Type	Main Topic	Principal Findings
Ferrero et al. [5]	2013	Prospective surgical study	IOUS in CRLM surgery	IOUS continued to modify operative strategy despite modern preoperative imaging
Jarnagin et al. [6]	2015	Retrospective cohort	Intraoperative staging	IOUS improved detection of occult hepatic lesions and vascular relationships
Cohen et al. [7]	2005	Clinical observational study	Hepatic lesion detection	IOUS identified additional malignant lesions not detected preoperatively
Sietses et al. [8]	2010	Comparative study	IOUS vs. preoperative imaging	Intraoperative ultrasonography significantly altered resection planning
Wagnetz et al. [60]	2011	Comparative imaging study	MRI vs. IOUS	IOUS remained complementary to high-resolution MRI and CT
Takahashi et al. [20]	2012	Clinical study	CE-IOUS using perfluorobutane	Improved intraoperative detection and characterization of CRLM
Hoareau et al. [18]	2016	Prospective study	CE-IOUS in CRLM	CE-IOUS improved identification of occult lesions after chemotherapy
Bitterer et al. [19]	2025	Clinical review	CE-IOUS in hepatic oncology	Enhanced diagnostic sensitivity for HCC and CRLM
Russolillo et al. [22]	2021	Comparative study	Laparoscopic IOUS	Laparoscopic ultrasound remained valuable despite liver-specific MRI
Torzilli et al. [28]	2008	Surgical series	Parenchymal-sparing hepatectomy	IOUS-guided vascular-oriented resections preserved functional liver parenchyma
Arita et al. [29]	2011	Clinical study	Anatomical liver resection	IOUS facilitated individualized segmental hepatectomy planning
Oba et al. [25]	2018	DLM management study	Disappearing liver metastases	Residual viable tumor frequently persisted despite radiological disappearance

The table summarizes landmark clinical studies that significantly contributed to the evolution of intraoperative ultrasound and contrast-enhanced intraoperative ultrasound in hepatic oncology surgery, particularly regarding lesion detection, intraoperative staging, parenchymal-sparing hepatectomy, and precision-guided surgical strategies.

**Table 3:** Comparative imaging modalities in precision hepatic oncology surgery.

Imaging Modality	Main Advantages	Main Limitations	Best Clinical Applications
Computed Tomography (CT)	Wide availability; rapid acquisition; good vascular overview	Lower sensitivity for subcentimeter lesions and chemotherapy-treated metastases	Initial staging; assessment of vascular anatomy and extrahepatic disease
Magnetic Resonance Imaging (MRI)	Superior soft-tissue contrast; high sensitivity for CRLM; hepatocyte-specific contrast enhancement	Static preoperative assessment; limited intraoperative applicability	Detection of small liver metastases; characterization of indeterminate lesions
Transcutaneous Ultrasound (US)	Widely available; non-invasive; real-time imaging; no ionizing radiation	Operator-dependent; reduced sensitivity for small, deep, or obesity-associated lesions	Initial liver assessment; surveillance; image-guided biopsy
Contrast-Enhanced Ultrasound (CEUS)	Dynamic assessment of lesion vascularity; improved characterization of focal liver lesions; no ionizing radiation	Limited acoustic window; operator dependence; preoperative examination only	Characterization of indeterminate liver lesions; assessment after local ablative therapies; complementary imaging before surgery
Intraoperative Ultrasound (IOUS)	Real-time intraoperative imaging; dynamic vascular mapping; lesion localization during surgery	Operator-dependent; limited standardization	Intraoperative staging; anatomical guidance during liver resection
Contrast-Enhanced IOUS (CE-IOUS)	Improved lesion characterization and vascular perfusion assessment; enhanced detection of occult disease	Requires expertise and contrast administration; interpretation variability	Detection of residual disease; disappearing liver metastases; chemotherapy-treated liver
Indocyanine Green (ICG) Fluorescence Imaging	Real-time superficial tumor and biliary visualization; useful in minimally invasive surgery	Limited tissue penetration depth; false-positive fluorescence possible	Laparoscopic and robotic liver surgery; bile duct visualization
Three-Dimensional (3D) Reconstruction	Improved anatomical understanding and operative simulation	Preoperative static model; limited real-time adaptability	Surgical planning; volumetric assessment; complex anatomical resections
Navigation Systems and Augmented Reality (AR)	Enhanced spatial orientation; integration of preoperative and intraoperative imaging	High technical complexity; limited availability; registration inaccuracies	Precision-guided hepatic surgery; complex minimally invasive resections
Artificial Intelligence (AI)-Assisted Imaging	Automated image analysis; predictive modeling; potential real-time decision support	Experimental stage; requires validation and computational infrastructure	Future precision hepatic surgery and intelligent navigation systems

Comparative overview of imaging modalities currently integrated into modern precision hepatic oncology surgery, emphasizing their respective advantages, limitations, and principal clinical applications in hepatobiliary oncologic practice.

## Data Availability

No new data were generated or analyzed in this study. Data sharing is not applicable to this article.

## Abbreviations

The following abbreviations are used in this manuscript:

Abbreviation	Full Term
AI	Artificial Intelligence
AR	Augmented Reality
CE-IOUS	Contrast-Enhanced Intraoperative Ultrasound
CRLM	Colorectal Liver Metastases
CT	Computed Tomography
DLM	Disappearing Liver Metastases
HCC	Hepatocellular Carcinoma
ICG	Indocyanine Green
IOUS	Intraoperative Ultrasound
MRI	Magnetic Resonance Imaging
PET	Positron Emission Tomography
PRISMA	Preferred Reporting Items for Systematic Reviews and Meta-Analyses
rFA	Radiofrequency Ablation
US	Ultrasound
CEUS	Contrast-Enhanced Ultrasound
DCE-US	Dynamic Contrast-Enhanced Ultrasound
SERPS	Systematic Extended Right Posterior Sectionectomy
DLMs	Disappearing Liver Metastases
LI-RADS	Liver Imaging Reporting and Data System
3D	Three-Dimensional
WFUMB	World Federation for Ultrasound in Medicine and Biology
EFSUMB	European Federation of Societies for Ultrasound in Medicine and Biology
MDCT	Multidetector Computed Tomography
